# Not so Cool? Menthol’s discovered actions on the nicotinic receptor and its implications for nicotine addiction

**DOI:** 10.3389/fphar.2013.00095

**Published:** 2013-07-23

**Authors:** Nadine Kabbani

**Affiliations:** Department of Molecular Neuroscience, Krasnow Institute for Advanced Study, George Mason UniversityFairfax, VA, USA

**Keywords:** menthol, addiction research, African Americans, nicotinic receptors, tobacco

## Abstract

Nicotine cigarette smoke is a large public health burden worldwide, contributing to various types of disease. Anti-tobacco media campaigns and control programs have significantly reduced smoking in the United States, yet trends for menthol cigarette smoking have not been as promising. Menthol cigarette smoking is particularly prevalent among young adults and African Americans, with implications for long-term impacts on health care. Continuing high rates of menthol cigarette addiction call into question the role of menthol in nicotine addiction. To date, a biological basis for the high rate of addiction and relapse among menthol cigarette smokers has not been defined. Studies have demonstrated a role for menthol in the metabolism of nicotine in the body. More recent findings now reveal an interaction between menthol and the nicotinic acetylcholine (nACh) receptor in cells. This receptor is central to the actions of nicotine in the brain, and plays an important role in nicotine addiction. The newly discovered effect of menthol on nACh receptors may begin to explain the unique addictive properties of menthol cigarettes.

## BEYOND FLAVOR: MENTHOL’S SUCCESS AS AN ADDITIVE TO CIGARETTES

Menthol is a monocyclic terpene alcohol used widely as a flavoring in various pharmaceutical and commercial products (Eccles, 1994; Heck, 2010). It is also a common additive to tobacco cigarettes. The isomer *l*-menthol, which has been used as an additive in cigarettes since 1926, is extracted from the peppermint plant *Mentha arvensis. *The concentration of menthol in cigarettes varies by brand but is present in 90% of all tobacco products (Farco and Grundmann, 2013). It is used in menthol cigarettes to mask the harshness of smoke inhalation, increase the ease of smoking, and provide an oral sensation that appeals to many smokers. Today, menthol cigarettes account for 25% of the cigarette market in the United States (Heck, 2010; Hoffman and Simmons, 2011).

**FIGURE 1:**
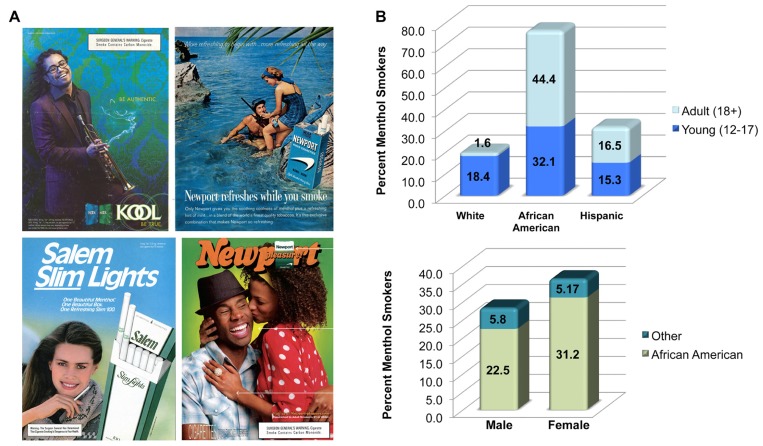
**Who smokes menthol cigarettes?(A)** Advertisements used in the marketing and branding of menthol cigarettes. **(B)** Data gathered from the Tobacco Products Scientific Advisory Committee’s original report (Wilson, 2011) on the percent menthol smokers in various ethnic, racial, gender, and age groups. Values are based on data obtained in 2008.

Targeted marketing strategies since the 1930’s have been effective in promoting smoking in young adults, women, and African Americans (Rosenbloom et al., 2012; Dauphinee et al., 2013; **Figure 1**). In the United States, the rate of menthol cigarette use has not receded even as the overall rate of smoking has declined in the general population. Young adults and teenagers in particular are more likely to try menthol cigarettes. Incidentally, young people who take up menthol cigarettes are 80% more likely to become life-long smokers than those who consume regular cigarettes (Friend, 2002; D’Silva et al., 2012). These findings present an important question for public health regulators and scientists: are menthol cigarettes more addictive than other cigarettes? Negative findings with respect to the impact of mentholated cigarettes on cancer, smoking initiation and other health issues exist (Rose and Behm, 2004; Werley et al., 2007; Hoffman and Miceli, 2011^1^. In this article, I explore how recent findings on interactions between menthol and nicotinic acetylcholine (nACh) receptors can shed new light on the addictive potential of menthol cigarettes.

## DOES MENTHOL CONTRIBUTE TO ADDICTION?

Traditionally, research into the addictive properties of tobacco cigarettes has focused on the actions of nicotine in the brain. The role of menthol has been attracting increasing attention, and emerging research suggests that menthol’s role in tobacco addiction deserves even closer scrutiny. One of the more significant scientific suggestions is that menthol can alter the metabolism of nicotine in the body. This effect of menthol is shown to provide smokers an enhanced exposure to systemic nicotine and its metabolites (Benowitz, 2008). In accordance with this idea, menthol cigarette smokers on the whole are known to exhibit different smoking habits from other smokers. For example, they tend to smoke fewer cigarettes per day, and find it more difficult to quit (Oliver et al., 2013). Relapse rates are also considerably higher in menthol cigarette smokers suggesting that menthol may contribute more than just flavor.

Menthol is known to directly activate the cold sensitive transient receptor potential melastatin (TRPM) ion channel in cells, which contributes to the cool nasal and oral sensation provided by menthol. Additionally, menthol has been shown to depress respiration and enhance nicotine’s presence in the lungs (Janssens and Voets, 2011). How the mechanisms of menthol’s actions impact nicotine addiction, on the other hand, is not clear.

## WAYS IN WHICH MENTHOL CAN CONTRIBUTE TO ADDICTION

### MENTHOL ALTERS NICOTINE METABOLISM

A number of scientific studies show that menthol inhibits the metabolism of nicotine, thereby enhancing nicotine delivery via menthol cigarettes (Benowitz, 2008). Smoking menthol cigarettes appears to also increase systemic exposure to tobacco smoke toxins and other toxins such as carbon monoxide, possibly by affecting the metabolism of nicotine (Hoffman, 2011). Differences in nicotine metabolism prevail in the general population. These can be accounted for by genetic as well as environmental factors. African-Americans, in particular, appear to metabolize nicotine to its main metabolite cotinine more slowly, and to metabolize cotinine itself more slowly (Benowitz et al., 2011). Whether genetic and environmental factors also predispose individuals to an altered response to menthol is interesting to consider. A number of reviews have been published on this topic (Benowitz, 2009; Ahijevych and Garrett, 2010; Hoffman and Simmons, 2011), and therefore it will not be discussed in this article.

### MENTHOL INTERACTS WITH THE NICOTINIC RECEPTOR

While nicotine is considered to mediate most of the pharmacological and addictive properties of tobacco via the actions of the nACh receptor in the brain (Albuquerque et al., 2009; Berrettini and Doyle, 2012; Picciotto and Kenny, 2013), a new line of evidence suggests that menthol also regulates nACh receptor activity (Ahijevych and Garrett, 2010; Fagan et al., 2010; Hoffman and Simmons, 2011). During the past year, work from various labs has shown that menthol can directly modulate the pharmacological actions of nicotine on the nACh receptor. In doing so, menthol is found to alter the effect of nicotine in neural cells (Hans et al., 2012; Ashoor et al., 2013). An additional new study reveals that the density of nACh receptors in the brain of menthol cigarette smokers is considerably higher than that of non-menthol smokers (Brody et al., 2013). Taken together, these emergent findings suggest a new framework for understanding the role of menthol in nicotine addiction.

#### Menthol directly regulates the actions of the nicotinic receptor in the cell

Prolonged cigarette smoking is associated with an increase in nACh receptor density throughout the brain (Picciotto and Kenny, 2013). This process of receptor “up-regulation” is likely to play an important role in the physiological underpinnings of nicotine addiction. A similar mechanism of receptor up-regulation occurs in rodents in response to chronic nicotine administration (Le Foll and Goldberg, 2009). This process accompanies a propensity to self-administer nicotine (Moretti et al., 2010) thus suggesting that these alterations in nACh receptor levels contribute to nicotine addiction. A new study by Brody et al. (2013) shows that smokers of menthol cigarettes also exhibit an up-regulation in nACh receptors throughout the brain. The up-regulation in nACh receptor levels is significantly higher, however, in smokers of menthol cigarettes than other types of cigarettes. Using positron emission tomography (PET) to visualize the density of α4β2 nACh receptors, the authors find that menthol smoking is associated with a 9–28% rise in the levels of these nACh receptors in regions such as the prefrontal cortex and corpus callosum.

Two other studies published this year by Hans et al. (2012) and Ashoor et al. (2013) highlight a shared mechanism of action for menthol on nACh receptors. These studies show that menthol directly attenuates the activation of the nACh receptor by nicotine. Single channel recordings, combined with whole cell patch clamp analysis, reveal that menthol decreases the ability of nicotine to activate the nACh receptor. In the case of the α4β2 nACh receptor, interaction with menthol appears to be most favorable when the receptor is in the closed state (Hans et al., 2012). Similar findings are reported for the α7 nACh receptor, which demonstrate that menthol inhibits the responsiveness of the receptor to nicotine in cells (Ashoor et al., 2013). The amount of menthol used in the above experiments is consistent with one report on the levels of menthol detected in the brain tissue of mice (Pan et al., 2012). Together, these studies corroborate menthol’s ability to regulate nACh receptors in the brain^2^.

**FIGURE 2:**
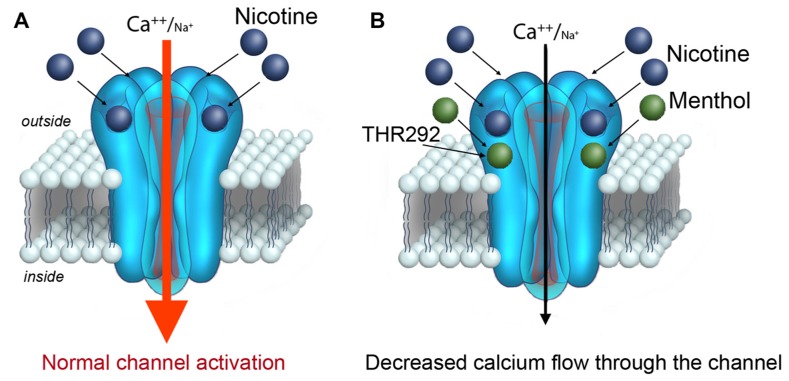
**A role for menthol in modulating the activation of the α7 nACh receptor**.(A) Nicotine is a known to bind and activate the α7 nACh receptor channel at the plasma membrane. Upon binding nicotine, the nACh receptor channel opens conducting calcium (and sodium) into the cell. **(B)** New findings by Ashoor et al. (2013) reveal that nicotine does not activate the α7 nACh receptor channel to the same extent when co-applied with menthol. Similar findings have also been reported for α4β2 nACh receptors (Hans et al., 2012). Threonine 252 within the α7 nACh receptor has been suggested to contribute to menthol binding.

Structural modeling based on sequence homology and docking simulations was also provided in Ashoor et al. (2013). The computational perspective suggests two energetically favorable residues within the nACh receptor protein for menthol binding. The proximity of the two residues within the nACh receptor implies a possible menthol-binding site within the nACh receptor channel. Interestingly, this putative binding site appears well outside of the nicotine-binding pocket, and suggests that menthol can function as an allosteric modulator of the nACh receptor (Ashoor et al., 2013; **Figure 2**). The structural modeling data supports experimental evidence on the actions of menthol on the nACh receptor. In particular, studies show that menthol attenuates the nACh receptor channel response to nicotine in a dose-dependent, non-competitive manner, and does not interfere with the kinetics of the nACh receptor channel (Hans et al., 2012; Ashoor et al., 2013). Taken together, the findings prove that menthol modifies the responsiveness of the nACh receptor to nicotine, thereby potentially affecting nicotine’s action in the brain.

#### Menthol alters the addictive properties of nicotine

Nicotinic receptors play a key role in addiction by regulating the release of several important neurotransmitters in the brain (Picciotto and Kenny, 2013). Nicotine directly enhances dopamine levels in the mesolimbic system by interacting with nACh receptors on dopaminergic neurons in the ventral tegmental area (VTA). Nicotine can also regulate the release of glutamate and GABA by binding to nACh receptors within the VTA (Grabus et al., 2005; Maskos, 2008). The expression of nACh receptors in regions such as the nucleus accumbens, hippocampus, amygdala, and prefrontal cortex enables nicotine to influence brain functions that contribute to addiction, including memory and decision making. These receptors are also present on neurons that release other neurotransmitters, including opioids, norepinephrine, serotonin, and cannabinoids (Maskos, 2008). However, the role of these neurotransmitters in nicotine dependence is not clear.

Strong preclinical evidence suggests that β2, α4, α5, α6, and α7 containing nAChRs mediate the reinforcing effects of nicotine (Changeux, 2010). A genetic variation in the human α7 nACh receptor gene CHRNA7 has also been linked to tobacco dependence (Albuquerque et al., 2009; Berrettini and Doyle, 2012). Mice with a deletion of the α7, α4, or β2 gene are viable, but exhibit altered responses to nicotine (Stoker and Markou, 2013). In behavioral studies, activation of α7 as well as α4β2 nACh receptors strongly correlates with an animal’s propensity to self-administer nicotine (Le Foll and Goldberg, 2009). Thus, if menthol modulates the function of these nACh receptors, it is plausible that it also modifies the addictive properties of nicotine in the brain. In one scenario, menthol can directly modulate the actions of nicotine on the α7nACh receptor as supported by findings in Ashoor et al. (2013). Electrophysiological data suggests that menthol attenuates the channel’s response to nicotine (**Figure 2**). At present however it is not certain that menthol does *not* alter the affinity of nicotine for the nACh receptor. Studies on the affect of menthol in α bungarotoxin binding to the α7 nACh receptor, suggest that menthol does not impact the binding of the receptor to this ligands (Ashoor et al., 2013). The findings, however, are based on patch-clamp experiments in oocytes and do not explore the effects of menthol on nACh receptor interaction with nicotine. The results therefore should be examined further in order to better understand how menthol modulates the activation of the nACh receptor by nicotine.

For the α7 nACh receptor channel, which opens with a strong inward depolarizing calcium (and sodium) current in response to nicotine (Albuquerque et al., 2009), simultaneous exposure to menthol is predicted to attenuate the channel response (**Figure 2**). Mechanistically, menthol can do this by potentiating the desensitization of the channel, or by inhibiting its full activation by the agonist. In either case, the short-term effect of weakening the α7 nACh receptor signal in neurons appears to correlate with a state of craving and an increase in motivation to smoke (as discussed below; Brunzell and McIntosh, 2012). In the long-term, menthol may promote up-regulation in nACh receptors in the brain. This is supported by the finding in Brody et al. (2013), which indicate higher levels of α4β2 receptors in the brain of menthol cigarette smokers relative to non-menthol smokers. The effects of menthol on nACh receptor expression may play a role in addiction to nicotine as well as in the difficulty to quit smoking menthol cigarettes.

How menthol can engage the brain’s reward pathway is an important scientific question to consider. The role of menthol cigarettes in increasing the reinforcing effects of nicotine has been recently explored in rodents (Abobo et al., 2012). Menthol has been found to alter the dopamine system in rodents by interacting with the dopamine uptake inhibitor bupropion (Umezu and Morita, 2003). Future studies in rodents, as well as humans, should shed light on the relevance and properties of the interaction between nACh receptors and menthol. Because a decrease in α7 nACh receptor function appears sufficient to alter the motivation to smoke, increases nicotine self-administration, and alter the function of the dopamine system in rodents (Brunzell and McIntosh, 2012), it is plausible that menthol can contribute to addiction by attenuating the nACh receptor.

## CONCLUSION

Recent findings on the ability of menthol to modulate nACh receptors suggest an important role for this compound in smoking addiction. While compelling, these studies are not yet conclusive. Rather, these findings point to the need for more studies to determine if and how menthol affects the activity of nACh receptors in the brain. Experiments using subunit specific nACh receptor knockout mice are a clear next step in the pharmacological and behavioral analysis of menthol function. *Ex vivo* studies in cultured cells and brain slices are also important to confirm and characterize interactions between menthol and the nACh receptor. For example, studies that examine menthol’s effects on nicotine self-administration in rodents will enable the assessment of dopamine (and glutamate release) in the brain following the administration of nicotine or nicotine with menthol. Subunit specific knockout mice for the α4,βb2,and α7 subunits as well as other nACh receptors (Changeux, 2010), will permit testing of menthol’s effect on various types of receptors. Lastly, genetic testing for polymorphisms in nACh receptor genes in smokers may provide clues on why some people are more prone to menthol cigarette addiction. Findings on genetic variability in nicotine metabolism in African Americans clearly suggest a role for genes in (nicotine) addiction (Benowitz et al., 2011). Recent work has also linked variants in genes encoding the α3, α5, and β4 nACh receptor subunits to smoking risk in the general population (Wang et al., 2012). If menthol were indeed found to impact the function of nACh receptors, a clear discussion would then be warranted about the addictive properties and public health implications of menthol nicotine cigarettes.

## Conflict of interest Statement

The author declares that the research was conducted in the absence of any commercial or financial relationships that could be construed as a potential conflict of interest.

## Footnotes

1 The study was supported by tobacco industry funding.

2 Liquid chromatography measures of free menthol in the brain of mice after a single intraperitoneal injection of menthol (100 mg/Kg), indicate 54.6 µg/g and 5 µg/g at 5 and 60 min after injection, respectively (Pan et al., 2012). Calculations based on an average adult mouse weight suggest that the levels of menthol in the brain are between 30 and 350 µM
